# Bromide Rashes

**Published:** 1907-07-27

**Authors:** 


### BROMIDE RASHES.

A male infant of eight months was brought last week to a skin clinic with a peculiar pustular eruption upon the left arm and thigh. The mother stated that the child was vaccinated at the usual time and that all had gone well until two months ago, when she noticed that the vaccination scars "began to look funny " as if they were " coming up again." The public vaccinator, to whom she took the child as having performed the operation, prescribed some dusting powder, but matters did not improve; in fact, some fresh places appeared upon the thigh. On examination the rash was seen to consist of isolated pustules, which, in two or three places, had coalesced to form a rounded, elevated plaque, dusky pink in colour, with practically no surrounding erythema. The individual lesions, if seen upon the face of an adult, would have passed for a severe pustular acne. Having recognised the condition, a careful inquiry was made with a view of tracing, if possible, the administration of the drug, but this was not successful, for although the child was attending the casualty department of the hospital for whooping-cough and was receiving medicine which might have contained bromide, yet the mother persisted in the statement that the rash had appeared before this. The infant was being nursed and the mother had not taken any medicine herself.

The common acneiform type of bromoderma, the so-called " bromic acne," is familiar enough, especially among epileptics. The variety, originally described by Cholmely, known as " confluent acne," is that usually seen in infants. These lesions, if unrecognised, may go on to become fungating or fleshy growths, and they are very puzzling to those who are not familiar with the condition. When ulceration occurs the eruption has even been mistaken for sarcoma; but the greatest similarity is, perhaps, to multiple vaccinia. It is noteworthy that the general health is rarely affected and both pain and itching are conspicuous by their absence. In some cases the breath may smell of bromine, and the urine may give a positive test for bromides. The appearance of the rash is determined by the dose of the drug, the duration of administration, and by the susceptibility of the patient. A single teething powder may, in a suitable case, induce the eruption. It is not even necessary that the bromide should be given directly to the infant, for many observers have reported cases in which the eruption has appeared in babies nursed by mothers who were themselves taking the drug.

In most cases it is sufficient to stop all medicines, powders, etc., which are known to contain bromide. If the disappearance of the eruption be tardy a minute dose of Fowler's solution, as recommended by Gowers for bromine-acne, will be useful in hastening the process. The eliminative organs must, at the same time, be encouraged to act well.

